# IL-7 Promotes CD95-Induced Apoptosis in B Cells via the IFN-γ/STAT1 Pathway

**DOI:** 10.1371/journal.pone.0028629

**Published:** 2011-12-14

**Authors:** Stefano Sammicheli, Linh Dang Vu Phuong, Nicolas Ruffin, Thang Pham Hong, Rebecka Lantto, Nancy Vivar, Francesca Chiodi, Bence Rethi

**Affiliations:** Department of Microbiology, Tumor and Cell Biology, Karolinska Institutet, Stockholm, Sweden; MRC National Institute for Medical Research, United Kingdom

## Abstract

Interleukin-7 (IL-7) concentrations are increased in the blood of CD4+ T cell depleted individuals, including HIV-1 infected patients. High IL-7 levels might stimulate T cell activation and, as we have shown earlier, IL-7 can prime resting T cell to CD95 induced apoptosis as well. HIV-1 infection leads to B cell abnormalities including increased apoptosis via the CD95 (Fas) death receptor pathway and loss of memory B cells. Peripheral B cells are not sensitive for IL-7, due to the lack of IL-7Ra expression on their surface; however, here we demonstrate that high IL-7 concentration can prime resting B cells to CD95-mediated apoptosis via an indirect mechanism. T cells cultured with IL-7 induced high CD95 expression on resting B cells together with an increased sensitivity to CD95 mediated apoptosis. As the mediator molecule responsible for B cell priming to CD95 mediated apoptosis we identified the cytokine IFN-γ that T cells secreted in high amounts in response to IL-7. These results suggest that the lymphopenia induced cytokine IL-7 can contribute to the increased B cell apoptosis observed in HIV-1 infected individuals.

## Introduction

Interleukin-7 acts as a survival factor for resting peripheral T cells via the maintenance of cellular homeostasis and by promoting the expression of anti-apoptotic proteins. In addition, IL-7 can serve as a costimulatory factor during T cell activation, a role that is particularly important in conditions associated with lymphopenia when IL-7 triggers hoemostatic proliferation. The progressive loss of peripheral T lymphocytes in HIV-1 infected individuals, as well as in other lymphopenic conditions, has been associated with increased concentrations of IL-7, and higher IL-7 levels could possibly contribute to the accelerated T cell activation [1], [2], [3]. Although the potential effects of the lymphopenia induced higher IL-7 levels remain speculative, animal models indicated that the modulation of IL-7 levels has a strong impact on T cell homeostasis. Increased T cell numbers and signs of autoimmunity were detected at higher IL-7 doses, whereas a higher competition for IL-7 via IL-7Rα overexpression led to decreased T cell numbers [4], [5], [6], [7], [8].

In our previous studies we showed that the IL-7 induced T cell stimulation can lead to increased sensitivity to activation induced apoptosis via the CD95 death receptors [9], [10]. IL-7 increased the expression of the CD95 on resting T cells, induced a polarized cell surface distribution of the molecule and increased the sensitivity of T cells to CD95 mediated apoptosis. Serum IL-7 levels correlated with CD95 expression and apoptosis sensitivity of T cells in HIV-1 infected patients further indicating a potential link between high IL-7 levels and increased T cell apoptosis in lymphopenic conditions [9].

Normally, CD95 molecules play an important role in regulating T and B cell homeostasis. Activated T and B lymphocytes both up-regulate CD95 expression and require anti-apoptotic signals to escape CD95 mediated apoptosis. Among B cells, the ones receiving strong BCR signals via high affinity antigen recognitions are able to avoid CD95-induced apoptosis. On the other hand, weakly activated B cells or others receiving bystander T cell-derived signals only are likely to undergo apoptosis [11]. Based on this model, activation-induced sensitivity to CD95 mediated apoptosis might help the selection of B cells that carry high affinity antigen receptors during the course of B cell activation.

B cells are not the main targets of HIV-1 infection, but their defects have been described very early after the discovery of HIV-1 [12]. B cell subsets associated with immature, exhausted or activated apoptosis-prone phenotype accumulate in the circulation probably underlying decreased B cell functionality, increased B cell turnover and the loss of serological memory against pathogens [13], [14], [15]. The CD95 moelcules have been extensively implicated in both T and B cell apoptosis occurring in HIV-1 infected individuals. CD95 expression is elevated on T and B lymphocytes during HIV-1 infection, possibly as a consequence of the chronic and generalized immune-cell activation [13], [16], [17], [18], [19], [20], [21], [22]. The increased CD95 expression of B cells has been primarily associated with active viral replication during HIV-1 infection [13], however, ART does not lead to normalization of CD95 expression and B cell survival during chronic HIV-1 infection, indicating the presence of viremia-independent mechanisms that prime B cells to CD95-mediated apoptosis [22].

The lymphopenia induced cytokine IL-7 may not act directly on peripheral B cells due to the lack of IL-7Rα expression on these cells. On the other hand, high IL-7 levels have been associated with the accumulation of immature, transitional B cells in the circulation of lymphopenic patients indicating a potential impact of altered IL-7 levels on B cell homeostasis [23], [24].

In the present study we identified a novel regulatory mechanism that can lead to increased CD95 mediated B cell apoptosis in the presence of high IL-7 concentrations. IL-7 induced high CD95 expression on resting B cells and increased the sensitivity to CD95 mediated apoptosis via the cytokine IFN-γ that was produced by IL-7 treated T cells. These results indicate a potential link between high IL-7 levels and the increased B cell turnover during HIV-1 infection. In addition, our findings may also help to understand the events leading to decreased B cell numbers in patients receiving IL-7 therapy [25].

## Materials and Methods

### Cell culture

Blood samples were obtained from healthy blood donors (buffy coats). The ethical committee at the Karolinska Institutet approved our studies involving human samples. Peripheral blood mononuclear cells (PBMCs) were separated by Ficoll gradient centrifugation (Lymphoprep, Norway). For cell cultures, monocytes, T and B lymphocytes were separated using respectively the CD14 human microbeads, the Pan T cell Isolation Kit and B cells isolation kit II (Miltenyi Biotech, Bergisch Gladbach, Germany). The purity of the selected cell populations was 90–97% as measured by flow cytometry. Cells were cultured at a density of 1×10^6^ cells/ml in RPMI-1640 containing L-glutamine, 10% FCS and antibiotics. Human recombinant IL-2, IL-4, IL-7, IL-15 and IL-21 were all purchased from Peprotech (London, UK). The STAT1 inhibitor Fluodarabine was purchased from Sigma, St. Louis, MO. The neutralizing IFN-γ antibody was purchased from R&D Systems (Minneapolis, MN). For the generation of IL-7 treated T cell supernatants, T cells were cultured in 25 ng/ml IL-7 and new medium was added in every 2–3 days.

### Flow cytometric analysis

The following fluorochrome-conjugated antibodies were used: V450 BD Horizon anti-CD3 and anti-CD27, V500 anti-CD8, FITC anti-CD19, PE anti-CD127 (IL-7Rα a) and anti-CD95, PE-Cy5 anti-CD21, PE-Cy7 anti CD10 and anti CCR7, APC anti-IFN-γ and anti-CD95 (all from BD Pharmingen). PE-Texas Red-conjugated anti-CD4 was purchased from AbCam, PerCP-Cy5.5 anti CD19 and anti CD45RA were purchased from BioLegend and eBioscience respectively. Viable cells were detected using Near-IR-Live/Dead kit (Invitrogen). For the detection of STAT1 phosporylation, CD19+ B cells were sorted from PBMCs using a MoFlo XDP Cell Sorted (Beckman Coulter, CA) and cultured with IL-7 treated T cells supernatant or control supernatant. Phosphorylation of STAT1 was detected using an Alexa Fluor 647 anti-STAT1 (pY701) (BD Pharmingen). For the intracellular staining of IFN-γ T cells were separated from PBMCs and cultured for 5 days in the presence or absence of IL-7 and further activated with 50 ng/ml of phorbol myristate acetate (PMA) and 1 µM ionomycin (Sigma) for 6 hours in the presence of GolgiPlug (BD Pharmingen). Fluorescence intensities were measured with FACS LSR II (Becton Dickinson, CA) and data analysed with FlowJo v. 8.4.4 software (Tree Star Inc.).

### Apoptosis detection

CD95 mediated apoptosis was triggered by human recombinat CD95L (1 mg/ml), cross-linked with 20 mg/ml anti-His antibody (both from R&D System). FITC-conjugated Annexin V reagent (BD Pharmingen) was used to measure apoptosis according to manufacturer's instructions. The fraction of cells Vivid negative-Annexin V positive CD3 positive (T lymphocytes), or CD19 positive (B lymphocytes) were considered apoptotic.

### Protein Array

Sorted B cells were incubated in IL-7 treated or non treated T cell supernatants for 30 minutes and the phosphorylation patterns was determined using the Human Phospho-Kinase Array Kit (R&D Systems) according to manufacturer's instruction.

### Detection of IFN-γ mRNA levels in T cells

Cellular RNA was isolated from T cells using TriZol (Invritogen). Reverse transcription was performed with the High Capacity cDNA Archive kit (Applied Biosystems). For the real-time PCR we used human IFN-γ and the Transferrin Receptor or the Cyclophilin assays as endogenous controls, all from Applied Biosystems. The cycling reactions were performed with a 7900 CD95T ABI PRISM Sequence Detector System (Applied Biosystem).

### Measurement of cytokine concentrations

IFN-γ production was analysed in culture supernatants using ELISA (BD Pharmingen) according to manufacturer's recommendations.

## Results

### IL-7 treated T cells induce CD95 upregulation on B cells through the release of a soluble factor

**Figure 1:**
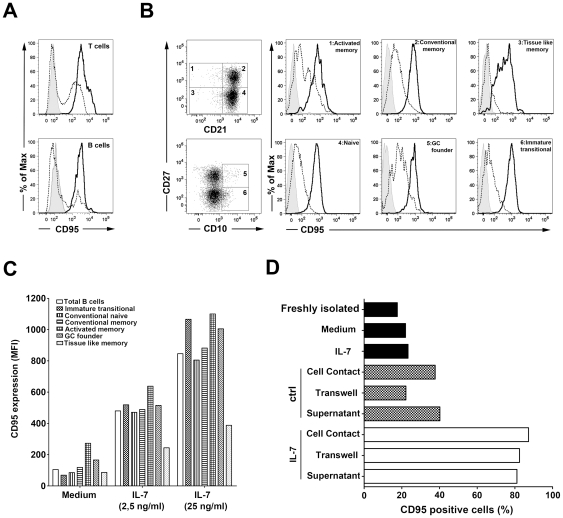
Induction of CD95 upregulation on B cells by IL-7 treated T cells. **A.** Representative histogram of CD95 expression among T and B lymphocytes from untreated (dashed line) or IL-7 treated (solid line) PBMCs cultured for 5 days. Grey filled histograms correspond to isotype control stainings. **B.** Peripheral B cell subsets were distinguished by measuring CD10, CD21 and CD27 expression. A representative CD95 staining on B cell subsets is shown after culturing PBMC for 5 days in presence (solid line) or absence (dashed line) of IL-7. Data are representative of 3 independent experiments. **C.** Cell surface levels of CD95 on different B cell subsets are shown measured after culturing PBMCs for 5 days in the presence of 25 or 2.5 ng/ml IL-7, or without the cytokine. D. Analysis of CD95 expression on freshly purified B cells or following a 3 day culture with and without IL-7. Alternatively, purified B cells were co-cultured with T cells using 1∶1 cell ratio allowing direct cell contact or separating the cells using a trans-well system. B cells were also plated in the presence of IL-7 treated or non treated T cell supernatants collected after culturing T cell for 5 days in the presence or absence of 25 ng/ml IL-7. Data are representative of 5 experiments.

We have previously shown that treatment of T cells *in vitro* with IL-7 upregulates CD95 expression and primes T cells to CD95 mediated apoptosis [9]. Interestingly, we observed that in IL-7 treated cultures of peripheral blood mononuclear cells (PBMC) B cells upregulated CD95 expression at a level comparable to T cells (Figure 1A). The IL-7 induced CD95 upregulation was similarly observed on several B cell subpopulations distinguished by the expression of CD10, CD21 and CD27 [15], namely CD21^+^CD27^−^ naïve, CD21^+^CD27^+^ memory, CD21^low^CD27^−^ tissue-like memory, CD21^low^CD27^+^ activated memory, CD10^+^CD27^−^ immature transitional, CD10^+^CD27^+^ germinal center-like B cells, as measured in PBMC cultured in presence of 25 ng/ml and 2.5 ng/ml IL-7 for five days (Figure 1B,C). The comparable upregulation of CD95 on different B cell subsets in presence of both high and low IL-7 concentrations indicates that these distinct B cell populations have similar sensitivity to IL-7 effects.

Importantly, mature B cells do not express the IL-7Rα subunit of the IL-7 receptor suggesting that IL-7 exerts its effect on B cells through an indirect mechanism. In order to better understand the mechanism leading to high CD95 expression on B cells in the presence of IL-7 we studied whether CD95 was induced by IL-7 directly or alternatively, by IL-7 treated T cells via the release of a B cell modulatory factor. B cells, isolated from peripheral blood of healthy donors did not upregulate CD95 in the presence of IL-7 during a 3-day culture (Figure 1D). On the other hand, in the presence of purified T cells, IL-7 efficiently induced CD95 expression on the majority of B cells. IL-7 treated T cells induced comparable CD95 expression on B cells in co-culture experiments or when the two cell types were separated in a trans-well system or when supernatant of an IL-7 treated T cell culture was added to B lymphocytes. These results indicated that IL-7 induced CD95 upregulation on B cells via a soluble mediator released by T cells. To further demonstrate the indirect nature of the IL-7 effect on CD95 expression of B cells, we treated purified B cells with the supernatants of IL-7 treated T cells for 3 days in the presence of 10 µg/ml neutralizing anti-IL-7 or isotype control antibodies. These experiments showed that IL-7 neutralization did not interfere with the ability of the IL-7 treated T cell supernatant to induce CD95 upregulation on B cells (data not shown).

### CD95 expression induced by IL-7 treatment of T cells increases the sensitivity of B cells to CD95 mediated apoptosis

**Figure 2:**
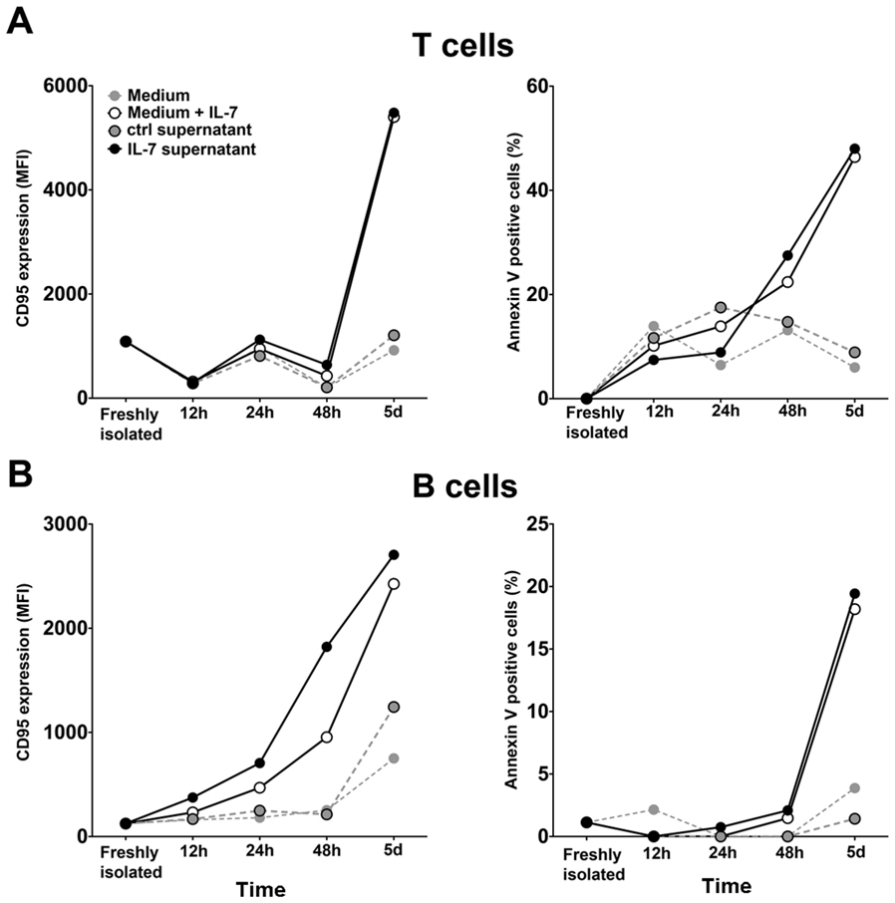
Enhanced CD95 mediated apoptosis of T and B cells upon IL-7 treatment. Kinetics of CD95 expression (left panels) and CD95 mediated apoptosis sensitivity (right panels) of CD3+ T cells (**A**) and CD19+ B cells (**B**) are shown, measured in PBMC cultures treated with 25 ng/ml IL-7, with supernatants of IL-7 treated or non treated T cell cultures or left untreated. Apoptosis was triggered using recombinant CD95L added for 24 hours to cultures. Data are representative of 3 independent experiments.

CD95 mediated apoptosis is regulated not only by CD95L availability but also by the expression level and membrane organization of the receptor and by the interaction of several pro- and anti-apoptotic factors influencing apoptotic signaling pathways [26]. Therefore, CD95 expression does not always correlate with sensitivity to CD95 mediated apoptosis. We studied whether the IL-7 induced high CD95 expression had an effect on B cell survival upon experimental CD95 triggering. We cultured PBMCs in the presence or absence of IL-7 for five days and monitored CD95 expression and sensitivity to CD95L induced apoptosis of both T and B lymphocytes at different time points. IL-7 induced a slow upregulation of CD95 expression on T lymphocytes accompanied by an increasing sensitivity of the cells to apoptosis triggered by CD95L (Figure 2A). B cells in the same cultures showed a similar, gradually increasing CD95 expression in response to IL-7 that resulted in an elevated sensitivity to CD95 mediated apoptosis at day 5 of the culture. IL-7, added to the PBMCs cultures, increased CD95 expression and CD95 mediated apoptosis with a similar kinetics as supernatants of T cells previously treated with IL-7 for five days. These results possibly indicate that the slow upregulation of CD95 on B cells is not the result of a delayed IL-7 effect on the CD95 inducing mediator molecule but rather a slow effect of the mediator itself. Our results showed that in the presence of high IL-7 concentration the upregulation of CD95 on B cells leads to a higher sensitivity to CD95 mediated apoptosis, suggesting a potential link between lymphopenia and B cell apoptosis.

### T cells treated with IL-7 induce STAT1 activation in B cells

**Figure 3:**
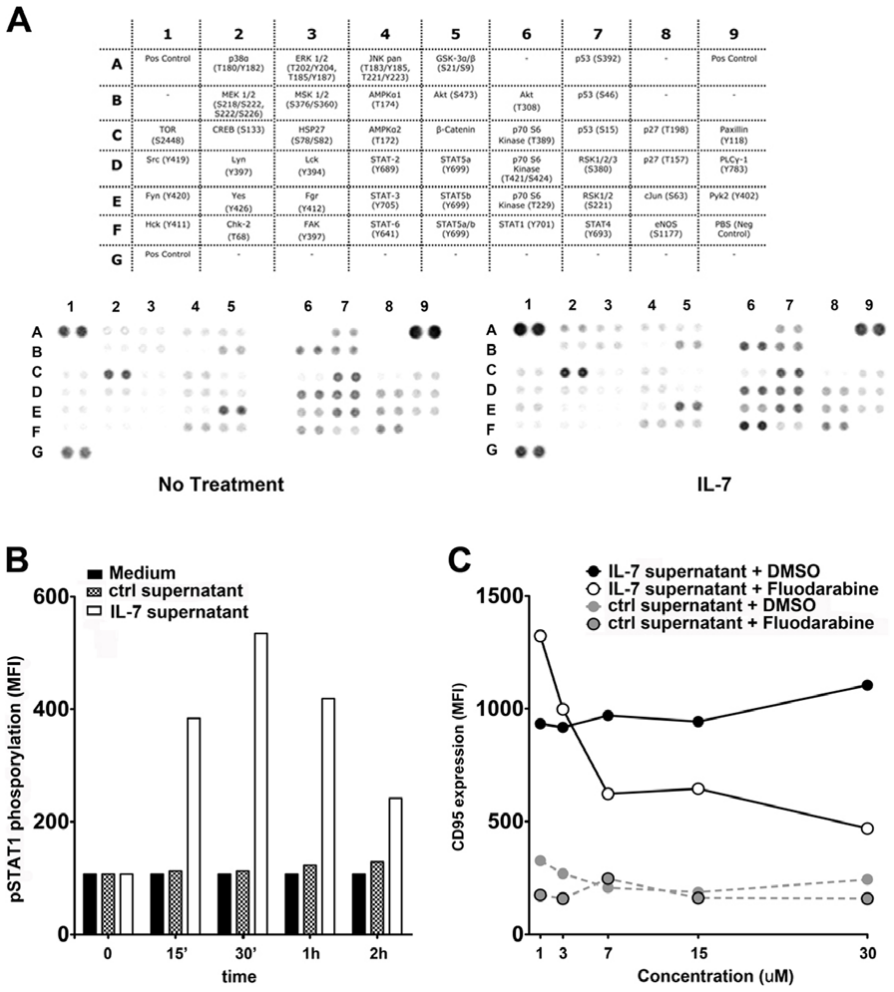
T cells treated with IL-7 induce STAT1 activation in B cells. **A.** B cells were purified from peripheral blood and were treated with supernatants of T cells cultured with or without IL-7 for 30 minutes at 37°C. In order to identify signaling pathways specifically induced by the IL-7 treated T cell supernatants we lysed the B cells and used a protein array to detect a wide range of intracellular phophorylation events (see table). Individual molecules are plotted in duplicates. **B.** STAT1 phosphorylation was studied in sorted B cells treated with supernatants of IL-7 treated or non treated T cells using flow cytometry. **C.** CD95 expression on B cells cultured in the presence of the STAT1 inhibitor fluodarabine or DMSO control in the presence of supernatants of IL-7 treated or untreated T cells. Data are representative of 3 independent experiments.

Expression of CD95 on lymphocytes is primarily a consequence of antigenic stimulation, and B cells can also express CD95 as result of CD40 engagement during a germinal center reaction [11]. Other signaling pathways leading to upregulation of CD95 on B cells are still elusive. In our setting, CD95 upregulation on B cells by IL-7 treated T cells was not affected by an antibody blocking CD40-CD40L interaction and T cells cultured for 5 days in the presence of IL-7 remained negative for CD40L suggesting that IL-7-induced CD95 expression is independent of CD40 activation (data not shown). In order to identify the IL-7 induced modulatory factor acting on B cells first we performed a molecular weight-based filtering of the IL-7 treated T cell supernatants and found that the factor inducing CD95 expression on B cells was present in the MW range between 30–100 KD, thus excluding T cell-derived exosomes or membrane fragments as mediators for such activity (data not shown). Next we analyzed signaling pathways induced in B cells by supernatants of IL-7 treated T cells using a protein array detecting 46 individual phophorylation events on different signaling components. Sorted B cells were incubated in IL-7 treated or non-treated T cell supernatants for 30 minutes and thereafter the phosphorylation patterns induced in B cells were compared. We consistently observed an increased STAT1 phophorylation on residue Y701 in response to the IL-7 treated T cells supernatant (Figure 3A). In order to validate this protein array data we incubated sorted B cells with supernatant of IL-7 treated or non treated T cell cells and analyzed Y701 phophorylation of STAT1 using flow cytometry. As shown in figure 3B, IL-7 treated T cell supernatant induced a marked STAT1 phosphorylation in B cells whereas non treated T cell supernatants had no effect on STAT1 (Figure 3B). In order to evaluate the relevance of STAT1 activation in the increase of CD95 expression on B cells we cultured sorted B cells with IL-7 treated or non-treated T cell supernatant in the presence of Fluodarabine, a known STAT1 inhibitor [27]. As shown in Figure 3C, Fluodarabine decreased considerably the upregulation of CD95 on B cells in presence of IL-7 treated T cell supernatants, strongly suggesting that STAT1 serves as a signaling mediator of the IL-7 induced effects on B cells.

### IFN-γ acts as an IL-7 induced mediator that upregulates CD95 expression on B cells

STAT1 is posphorylated in response to both type I and II interferons; however, whereas type I IFNs induce signaling through STAT1/STAT-2 heterodimers, IFN-γ mediates its effects by inducing STAT1 homodimers [28], [29]. We did not detect phoporylation of STAT-2 or other STATs by protein array (Figure 3A) suggesting IFN-γ as a possible mediator of IL-7 effects on B cells. In addition, it has been shown that IFN-γ can induce CD95 expression on transformed cell lines of various origin and IL-7 treatment increased IFN-γ production by T cells triggered with anti-CD3 and anti-CD28 antibodies [30], [31], [32]. In order to test whether IL-7 induces IFN-γ production by resting T cells we measured IFN-γ concentrations in supernatants of IL-7 treated or non-treated T cell cultures.

**Figure 4:**
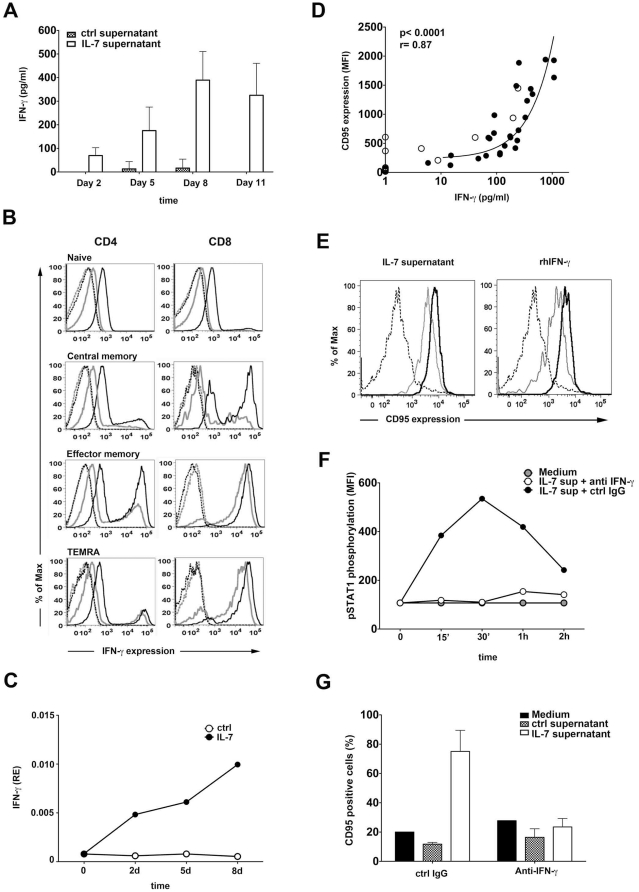
IL-7 induces CD95 upregulation on B cells via IFN-γ released from T cells. **A.** Purified T cells were cultured in the presence or absence of 25 ng/ml IL-7 and IFN-γ concentrations were measured at the indicated time points using ELISA. Mean values and standard deviations are calculated from the results of 7 independent experiments. **B.** IFN-γ expression was analyzed by flow cytometry in naïve (CD45RA+CCR7+), central memory (CD45RA-CCR7+), effector memory (CD45RA-CCR7−) and TEMRA (CD45RA+CCR7−) T cell subsets cultured in the presence (black line) or absence (grey line) of 25 ng/ml IL-7 for five days. Dashed lines represent isotype control staining. Data are representative of 3 independent experiments. **C.** IFN-γ mRNA levels were measured in IL-7 treated or untreated T cells by real time PCR. **D.** IFN-γ concentrations measured in the IL-7 treated or non-treated T cell supernatants are correlated with the ability of the same supernatants to induce CD95 expression on B cells (using the samples described in panel A). Black dots represent IL-7 treated, white dots represent non treated T cell supernatants collected at day 2, 5, 8 or 11. **E.** CD95 expression is shown on freshly isolated B cells (dashed line) or following a 12 h (grey lines) or 48 h (black lines) treatment with IL-7 treated T cell supernatants or 20 ng/ml recombinant human IFN-γ. **F.** STAT-1 phosphorylation is measured using flow cytometry in sorted B cells treated with supernatants of IL-7 treated T cells in combination with 10 mg/ml neutralizing anti-IFN-γ or isotype control antibodies. Data are representative of 3 experiments. **G.** B lymphocytes were cultured for five days with IL-7 treated or non treated T cell supernatants or were left untreated in the presence of 10 mg/ml neutralizing anti-IFN-γ or isotype control Abs. Thereafter the expression of CD95 was analyzed by flow cytometry. Data are representative of 3 different experiments.

Interestingly, IL-7 treatment induced the secretion of high levels of IFN-γ by T cells and the production remained stable during the 11 days of the experiment (Figure 4A). In non-treated T cell cultures we detected a measurable IFN-γ production in one donor out of the seven tested in this experiment. Notably, in our experiments IL-7 induced IFN-γ secretion in a resting T cell population, without concomitant TCR triggering. We detected similar amounts of IFN-γ produced by sorted CD4+ or CD8+ T cells in response to IL-7 treatment in three different experiments (data not shown). In order to identify the cellular source of IFN-γ in the IL-7 treated T cell cultures we studied IFN-γ production in CD4+ and CD8+ cells at various differentiation stages using intracellular flow cytometry. In non IL-7 treated T cell cultures we detected a small fraction of cells able to produce IFN-γ that were mostly memory cells (Figure 4B). Among CD8+ T cells the CD45RA-CCR7− and the CD45RA+CCR7− effector memory populations were enriched in IFN-γ producing cells whereas naïve, most CD4+ memory and CD8+ central memory T cells lacked the ability to produce IFN-γ. In cultures treated for 5 days with 25 ng/ml IL-7, the previously IFN-γ negative T cells obtained the ability to produce IFN-γ at a low but clearly detectable level, whereas T cells that already had the ability to secrete IFN-γ increased the production of the cytokine (Figure 4B). In addition, among memory subsets, we detected a shift from low IFN-γ producer to high IFN-γ producer profile, clearly detectable in CD4+ effector memory and CD8+ central memory T cells. IL-7 may thus not only provide an additive effect on IFN-γ production by raising weakly and evenly the ability of all T cells to secrete this cytokine, but it can induce high levels of IFN-γ released from cells previously lacking the ability of IFN-γ production. In line with the secretion of IFN-γ by IL-7 treated T cells we detected increased IFN-γ mRNA levels in IL-7 treated T cells suggesting that IL-7 increases IFN-γ transcription (Figure 4C).

Interestingly, the ability of supernatants collected from IL-7 treated and non-treated T cell cultures at the different time points (Figure 4A) to induce CD95 expression of B cells correlated with the IFN-γ concentration measured in the same samples (p<0.0001, Figure 4D). These results strongly suggested that IFN-γ, secreted by T cells, might act as a mediator of IL-7 activity on B cells. Indeed, culturing purified B cells with recombinant IFN-γ led to similar CD95 upregulation as observed in the presence of the IL-7 treated T cell supernatants (Figure 4E). In order to test whether the CD95 modulatory component in the supernatants of IL-7 treated T cells is IFN-γ, we cultured B cells with IL-7 treated T cell supernatants in presence or absence of IFN-γ neutralizing antibodies. IFN-γ neutralization efficiently blocked the ability of IL-7 treated T cell supernatants to induce STAT1 activation and CD95 upregulation in B cells (Figure 4F and G respectively). Taken together, these findings show that IFN-γ, induced by IL-7 treatment of T cells, would in turn act on B cells upregulating CD95 expression and increasing their sensitivity to CD95 mediated apoptosis.

### T cell mediated effect of the common γ-chain cytokines on CD95 expression of B cells

**Figure 5:**
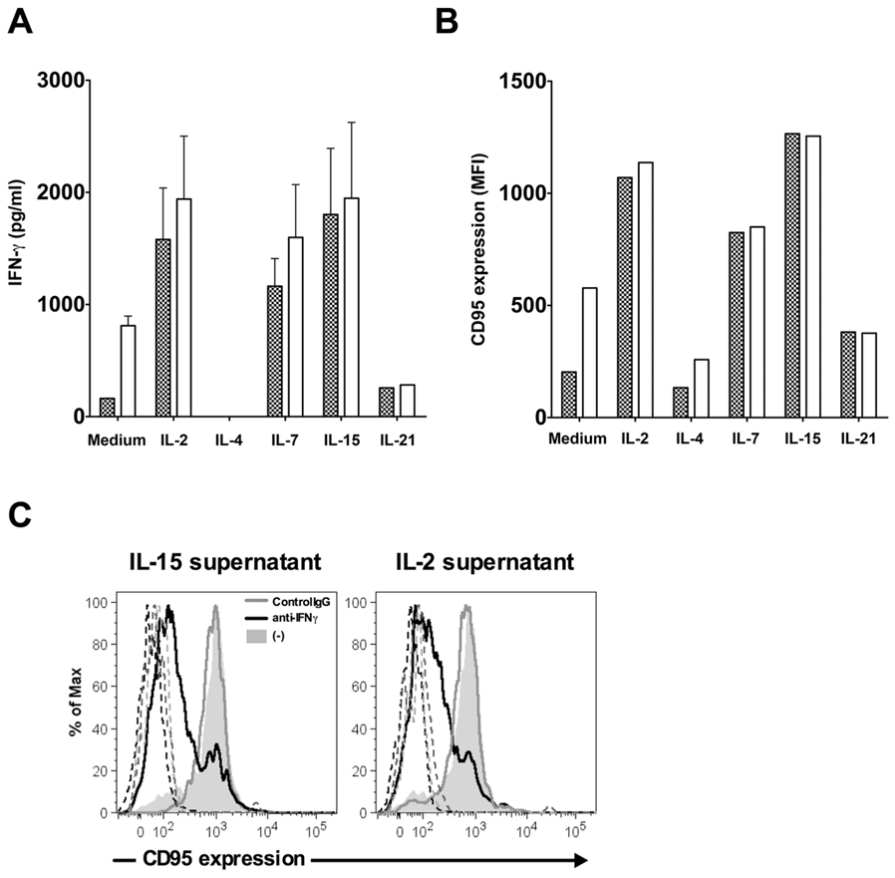
The effect of γ-chain cytokines on IFN-γ production by T cells. **A.** IFN-γ levels were measured in supernatants of T cells cultured in the presence of IL-2, IL-4, IL-7, IL-15 and IL-21 for five days. **B.** The same supernatants were tested for the ability to trigger CD95 upregulation on B lymphocytes. Representative data out of 3 independent experiments are shown. **C.** Purified B cells were cultured in the presence of IL-2 or IL-15 treated T cell supernatants for 3 days and then CD95 expression was analyzed by flow cytometry. IFN-γ was neutralized in the supernatants using 10 µg/ml anti-IFN-γ antibodies (black lines). Histograms with grey line represent samples containing isotype control antibody, the grey filled histograms represent samples without antibodies added and the dotted histograms represent stainings with isotype control antibody.

IL-7 signaling is transmitted by a receptor composed by the IL-7Rα together with the common γ-chain, a subunit shared by several cytokines, including IL-2, IL-4, IL-7, IL-15 and IL-21. Since IL-2 and IL-15 share the ability of IL-7 to induce CD95 expression on resting T cells [9], we tested whether the different γ-chain using cytokines could induce IFN-γ production by T cells. T cells were cultured for five days in the presence of the above mentioned γ-chain using cytokines and the culture supernatant was tested for IFN-γ concentration. Out of the five cytokines tested, supernatants of IL-2, IL-7 and IL-15 treated T cells contained high amount of IFN-γ (Figure 5A) and induced CD95 upregulation on B cells (Figure 5B), whereas IL-4 and IL-21 showed no such activity. In order to demonstrate that the IL-2 or IL-15 induced IFN-γ production could lead to CD95 upregulation on B cells we used neutralizing anti-IFN-γ antibodies and showed that IFN-γ neutralization strongly reduce CD95 upregulation on B cells induced by the IL-2 or IL-15 treated T cell supernatants (Figure 5C).

### Potential role of tissue environment on IL-7 induced IFN-γ production

**Figure 6:**
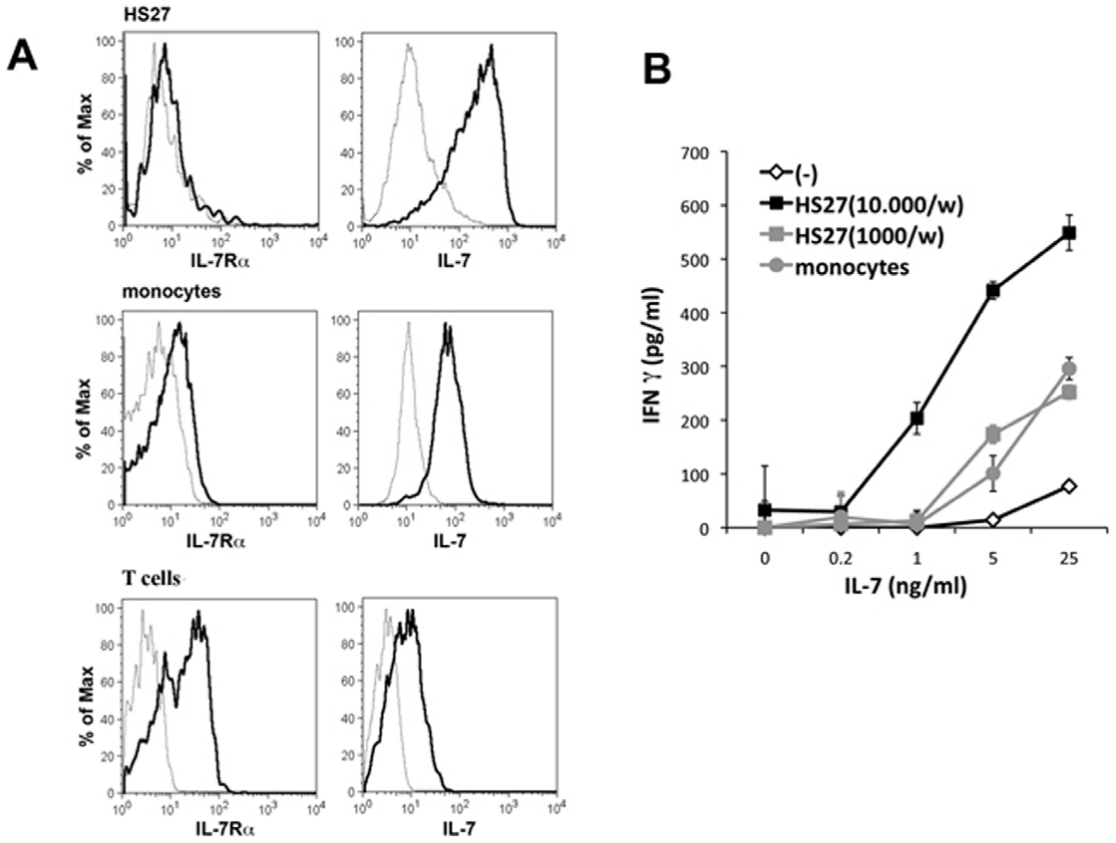
IL-7 presenting stroma cells increase IL-7 induced IFN-γ production in T cells. **A.** IL-7Rα expression and surface IL-7 binding was measured on the HS27 BM stromal cells as well as on CD14+ peripheral blood monocytes and CD3+ T cells using flow cytometry. **B.** The HS27 cell line (10000 or 1000 cells/well) or human monocytes (30000cells/well) were cultured in 96-well plates, pretreated with different concentrations of IL-7 for 2 hours at room temperature and thereafter purified peripheral blood T lymphocytes were added to the cultures for 3 days. IFN-γ production was measured after 3 days of culture using ELISA. Representative data of 3 independent experiments are shown.

It is predicted that T lymphocytes compete continuously for IL-7 and T cell numbers are sensitively regulated by the availability of the cytokine [33]. In addition to the concentration of IL-7, stromal cells able to concentrate this cytokine on the cell surface and deliver it to IL-7 sensitive cells may also have an impact on the efficiency of IL-7 on target cells [34], [35]. We tested whether the IL-7 induced IFN-γ production of T cells can be augmented by cell types that present IL-7 on the cell surface. We detected that the HS27 bone marrow derived cell line [36] bound high amounts of IL-7 in an IL-7Rα independent manner (Figure 6A). Peripheral blood monocytes also bound high amounts of IL-7; however these cells expressed detectable level of IL-7Rα indicating that the cytokine might simply be bound to its receptor. However, T cells in the same samples were characterised by a clearly higher IL-7Rα expression and by a similar or lower IL-7 binding ability as compared to monocytes, thus indicating that monocytes accumulate at least part of the cell surface associated IL-7 independently of the IL-7Rα molecules. The presence of the HS27 cell line had a strong effect on the IL-7 induced IFN-γ production in T cells leading to detectable IFN-γ production at otherwise insufficient IL-7 concentrations and greatly increased IFN-γ release at higher IL-7 doses (Figure 6B). Monocytes, albeit less efficiently than the bone marrow-derived stromal cells, could also increase IFN-γ production induced by IL-7. These results showed that the stimulatory effect of IL-7 on IFN-γ production is not only determined by the concentration of free IL-7, but that stromal cells surrounding T lymphocytes in the various tissues may also have a strong impact on IL-7 efficiency.

## Discussion

The cytokine IL-7 is often associated with increased T cell activation in T cell depleted individuals due to its stimulatory actions on survival and proliferation [37], [38]. A close link between CD4+ T cell depletion and increased blood IL-7 levels has been demonstrated in HIV-1 infection and in other, non-HIV related conditions of lymphopenia [1], [2], [23], [39] possibly reflecting the decreased IL-7 consumption associated with the loss of T cells. The persistent increase of IL-7 levels might stimulate a chronic and generalized T cell activation thus compensating the weakened T cell responses [3], [40]. On a long run, such non-antigen specific stimuli can lead to autoimmune diseases as demonstrated in IL-7 transgenic mice [6], [41].

Although it has been indicated that IL-7 production can be inducible in the liver by TLR ligands [42], steady state IL-7 production by the non-immune stromal cells requires no ongoing immune responses and the primary target cells for IL-7 are non-activated naïve and memory T cells [33]. Thus the source and the target cells clearly distinguish IL-7 from other γ-chain using cytokines that are typically produced during immune responses. Chronically elevated IL-7 levels in lymphopenic conditions may affect the majority of circulating peripheral lymphocytes in contrast to IL-2, IL-4, IL-15 or IL-21 that are released at sites of ongoing immune responses and act short term, on the population of activated lymphocytes only. The abilities of IL-7 to support peripheral T cell maintenance, contribute to homeostatic T cell expansion in addition to stimulating the production of new T lymphocytes made this cytokine an excellent candidate to improve peripheral T cell numbers in lymphopenic individuals [43].

IL-7 may not directly activate peripheral B cells, due to the lack of IL-7Rα expression on these. On the other hand, an intriguing correlation has been detected between IL-7 concentrations in human serum and the ratio of immature transitional B lymphocytes in HIV-1 infected individuals and in idiopathic CD4+ T cell lymphopenia [23], [24]. In addition, IL-7 therapy in humans led to transiently increased ratios of circulating transitional B cells [44], [45], [46]. Although the mechanism by which IL-7 leads to increased circulating levels of transitional B cells is yet to be clarified, the correlation between IL-7 levels and transitional B cell ratios indicates a potential influence of IL-7 on B cell homeostasis.

HIV-1 infection is associated with an increased sensitivity to activation-induced apoptosis among peripheral T cells, probably as a result of chronic and generalized activation of the immune system [21], [47]. The death receptors or ligands implicated in bystander apoptosis of non HIV-1 infected T cells are CD95 and TNF, induced by chronic T cell activation, and TRAIL, induced by plasmacytoid DC-derived IFN-alpha [48]. In our previous studies we showed that elevated IL-7 levels may not only support T cell activation during HIV-1 infection but can also contribute to T cell depletion through CD95 mediated apoptosis. We showed that CD95 expression, CD95 surface polarization and sensitivity to CD95 mediated apoptosis were all increased by IL-7 in resting peripheral T cells [9], [10]. The priming effect of IL-7 for CD95 mediated apoptosis might be particularly important in HIV-1 infected patients due the accelerated activation of both the adaptive and the innate immune system that could lead to increased Fas-L availability [49], [50].

Similarly to T cells, B cells also show an increased activation and apoptosis sensitivity during HIV-1 infection [13]. The increased sensitivity of B cells to apoptosis is accompanied by a decreased memory B cell ratios and loss of serological memory to pathogens [51], [52]. In our study we identified a novel mechanism through which IL-7 can increase CD95 expression and apoptosis sensitivity of peripheral B cells via the induction of IFN-γ in T cells. IFN-γ, released by IL-7 treated T cells, induced STAT1 phosporylation and CD95 upregulation in B cells. In addition, the IL-7 induced CD95 expression was associated with higher apoptosis sensitivity upon experimental CD95 ligation. These results indicate that the increasing IL-7 levels in HIV-1 infected individuals may contribute to CD95 mediated depletion of peripheral B lymphocytes. In line with this hypothesis, IL-7 therapy in humans resulted in a significant decline in peripheral B cell numbers that was reverted 1–2 weeks after cessation of the therapy [25]. Although the mechanism for such IL-7 induced B cell decline has yet not been clarified, whether it reflects redistribution or cell death, our results indicate the possibility that B cell apoptosis accelerated by high IL-7 levels could contribute to the decreased number of circulating B lymphocytes.

The effects of IL-7 on B cell apoptosis may be limited under steady-state conditions due to the low IL-7 levels. Similarly, the two other γ -chain cytokines, IL-2 and IL-15, that share the ability of IFN-γ induction with IL-7, may not act chronically and systematically but rather at sites of ongoing massive T cell activation or inflammation. The increasing levels of IL-7 in HIV-1 infected individuals may, on the other hand, strongly facilitate IFN-γ production particularly in tissue niches where the cytokine is produced and/or concentrated on the surface of stromal cells. Bone marrow, characterized by the presence of IL-7 producing stromal cells and memory T cells as well as secondary lymphoid tissues where lymphocytes migrate along an IL-7 producing fibroblast network may represent niches where IL-7 can influence B cell homeostasis. The fibroblast reticular cell network, however, might collapse in response to prolonged CD4+ T cell depletion and the IL-7 production in the lymphoid tissues might decline during the course of disease progression [53]. It is noteworthy that IFN-γ might increase the production of IL-7 by epithelial or bone marrow stromal cells [54] suggesting a positive regulatory loop when the IL-7 induced IFN-γ can further increase IL-7 production. However, further knowledge is clearly needed on the source of high IL-7 levels in order to better understand the localization, targets and effects of this cytokine in HIV-1 infected individuals.

The effects of IL-7 induced IFN-γ production among T cells might be multiple. Such mechanism may contribute to a better immunity at the price of less regulated and less localized T_H_1 type responses. On the other hand, when IFN-γ production is chronically supported and localized to IL-7 producing tissues or cells, rather than to sites of antigen-specific T cell activation, high IFN-γ levels may affect B cells independently of BCR triggering in the GC reactions. The IFN-γ induced CD95 upregulation on resting B cells may contribute to the increased apoptosis sensitivity of B cells observed in HIV-1 infected individuals and to the decreased maintenance of antigen-specific memory B cells.

Taken together, we presented a novel mechanism that implicates the T cell stimulatory environment associated with lymphopenia in bystander B cell apoptosis triggered by Fas activation.
